# The Role of the Reactive Oxygen Species and Oxidative Stress in the Pathomechanism of the Age-Related Ocular Diseases and Other Pathologies of the Anterior and Posterior Eye Segments in Adults

**DOI:** 10.1155/2016/3164734

**Published:** 2016-01-10

**Authors:** Małgorzata Nita, Andrzej Grzybowski

**Affiliations:** ^1^Domestic and Specialized Medicine Centre “Dilmed”, Ulica Bohaterów Monte Cassino 3, 40-231 Katowice, Poland; ^2^Department of Ophthalmology, Poznan City Hospital, Ulica Szwajcarska 3, 61-285 Poznań, Poland; ^3^Chair of Ophthalmology, Medical Faculty, University of Warmia and Mazury, Ulica Żołnierska 14 C, 10-719 Olsztyn, Poland

## Abstract

The reactive oxygen species (ROS) form under normal physiological conditions and may have both beneficial and harmful role. We search the literature and current knowledge in the aspect of ROS participation in the pathogenesis of anterior and posterior eye segment diseases in adults. ROS take part in the pathogenesis of keratoconus, Fuchs endothelial corneal dystrophy, and granular corneal dystrophy type 2, stimulating apoptosis of corneal cells. ROS play a role in the pathogenesis of glaucoma stimulating apoptotic and inflammatory pathways on the level of the trabecular meshwork and promoting retinal ganglion cells apoptosis and glial dysfunction in the posterior eye segment. ROS play a role in the pathogenesis of Leber's hereditary optic neuropathy and traumatic optic neuropathy. ROS induce apoptosis of human lens epithelial cells. ROS promote apoptosis of vascular and neuronal cells and stimulate inflammation and pathological angiogenesis in the course of diabetic retinopathy. ROS are associated with the pathophysiological parainflammation and autophagy process in the course of the age-related macular degeneration.

## 1. Introduction

The reactive oxygen species (ROS) form as products under normal physiological conditions due to the partial reduction of molecular oxygen. ROS, that is, superoxide anion (O_2_^−^), hydroxyl radical (OH^•^), hydrogen peroxide (H_2_O_2_), and singlet oxygen (^1^O_2_), arise in many ways, as a product of the respiratory chain in mitochondria, in photochemical and enzymatic reactions, as a result of the exposure to UV light, ionizing radiation, or heavy metal ions. Superoxide is generated directly from the reduction of oxygen and then dismutated to hydrogen peroxide. Hydrogen peroxide is a molecule with low reactivity, but it can readily penetrate cell's membranes and generate the most reactive form of oxygen, the hydroxyl radical, via Fenton's reaction (H_2_O_2_ + Fe^2+^ → Fe^3+^ + OH^−^ + OH^•^).

Low levels of ROS production are required to maintain physiological functions, including proliferation, host defense, signal transduction, and gene expression [1]. ROS are produced mainly by mitochondrion. The mitochondrial electron transport chain generates superoxide radicals through the single-electron leak at respiratory complexes I and III of the oxidative phosphorylation (OXPHOS) pathway [1, 2]; however, flavin-dependent enzymes in the mitochondrial matrix may produce the reactive oxygen species at much higher rates than complex I [3]. Under physiological conditions there is a cellular balance between ROS generation and clearance, since eukaryotic cells have several antioxidative defense mechanisms, including enzymes and antioxidants. There are five major types of primary intracellular antioxidant enzymes, that is, Cu/Zn-superoxide dismutase (Cu/Zn-SOD, SOD1) in the cytosol, manganese superoxide dismutase (Mn-SOD, SOD2) in the mitochondrial matrix, catalase, glutathione peroxidase (GPx), and glutathione reductase (GR). The SODs dismute superoxide to oxygen and hydrogen peroxide, while catalase and GPx convert hydrogen peroxide into H_2_O and O_2_. Apart from the antioxidant enzymes, small molecular weight and nonenzymatic antioxidants are also involved in the protection of the intracellular components against the reactive oxygen species. However, when ROS cellular overproduction overwhelms intrinsic antioxidant capacity, then the oxidative stress occurs and next the damage to the biomolecules of normal cells and tissues may occur [1].

The oxidative stress usually results from either excessive ROS production, mitochondrial dysfunction, impaired antioxidant system, or a combination of these factors. The prooxidative/antioxidative cellular imbalance between the ROS production and ability of the biological systems' defense mechanisms to eliminate the cellular stress disturbances leads to the vicious circle, since the oxidative stress reciprocally aggravates ROS production. ROS can be generated at elevated rates under normal aging, as well as in acute or chronic pathophysiological conditions [4–6]. The excessiveness of ROS causes oxidative damage to the deoxyribonucleic acid (DNA), proteins, and lipids. ROS can react with the nucleic acids attacking the nitrogenous bases and the sugar phosphate backbone and can evoke single- and double-stranded DNA breaks. Human mitochondrial DNA (mtDNA) is a covalently closed, double-stranded molecule, encoding 13 proteins of the oxidative phosphorylation chain, 22 tRNAs, and 2 rRNAs. mtDNA is more susceptible to the oxidative damage than its nuclear counterpart, since it is located in close vicinity to the inner mitochondrial membrane; a major site of ROS production is not protected by histones or other associated proteins, has intronless regions and a high transcription rate, and has a higher susceptibility to the oxidative modifications in its coding region. DNA damage induced by the oxidative stress may affect the protein-coding region of mtDNA and influence oxidative phosphorylation. mtDNA mutations can cause disturbances in the respiratory chain as well as the loss of control of ROS production. The much less effective repair system for mtDNA damage may be the cause for accumulating the oxidative stress together with its consequences. ROS also attack structural and enzymatic proteins by the oxidation of residual amino acids, prosthetic groups, formation of cross links, protein aggregates, and proteolysis. The inactivation of the key proteins can lead to the serious consequences in the vital metabolic pathways. Lipid peroxidation (autooxidation) is a process of oxidation of polyunsaturated fatty acids due to the presence of several double bonds in their structure and it involves production of peroxides (chemical compounds in which two oxygen atoms are linked together by a single covalent bond), ROS, and other reactive organic free radicals. There are several markers of oxidative damage, including the following: 8-hydroxy-2-deoxyguanosine (8-OHdG), a marker of oxidative damage to DNA; protein carbonyl groups, a marker of protein oxidation; malondialdehyde (MDA), a marker of lipid peroxidation; and 4-hydroxynonenal (4-HNE), a marker of lipid peroxidation [4–6].

The cell's inability to repair the incurred damage may cause genetically programmed cell's death (apoptosis) or mutations in the DNA, which leads to carcinogenesis or development of many neurodegenerative diseases. Increased ROS levels and oxidative damage of the cellular components play an important role in the senescence process. The amount of accumulated damage increases with the age due to impairs in the DNA repair system and the intensified oxidative stress as well as with the decreased antioxidant defense. Mutations in the key DNA repair genes result in an impaired recognition system and an inefficient repair of DNA damage, which accelerates the aging of the organism, leading to the age-related disruptions in cellular and tissue functions. The aging of the organism is an inevitable process since the formation of ROS is a result of normal daily cellular metabolism. Therefore, cells have complex defense mechanisms to combat both the formation of ROS and the impacts of their actions. The oxidative stress leads to apoptosis when antioxidant capacity is insufficient. The oxidative stress can induce apoptosis by damaging mtDNA, inhibiting the mitochondrial respiratory chain transition, and increasing mitochondrial membrane permeability [4, 7]. Cell's death induced by the excessive ROS production and the oxidative stress is involved in pathomechanism of many general neurodegenerative pathologies such as Alzheimer's disease [8], Parkinson's disease [9], prion disease [10], protein misfolding diseases [11], and ophthalmological diseases [12].

## 2. The Role of ROS and the Oxidative Stress in the Cornea Diseases

The cornea, an avascular tissue which maintains transparency at the frontal surface of the eye, contains three major layers, that is, the outer epithelium, a thick stroma with corneal fibroblasts, and the inner endothelium.

In the cornea, the source of oxidative stimuli is solar ultra violet (UV) radiation [13]; the human cornea absorbs 92% of UV-B, that is, 280–315 nm sunlight radiation reaching the eye [14] and atmospheric oxygen, mainly dioxygen [13]. Due to its localization and function, the cornea is chronically exposed to ROS accumulation as well as to the oxidative stress [13]; however, normal corneas have well-developed antioxidant defense systems which contain direct free radical scavengers, including nonenzymatic, low molecular weight antioxidants covering vitamin C, vitamin E, b-carotene [15, 16], reduced glutathione (GSH) [17], ferritin [18], *α*-tocopherol [19], and several indirect, enzymatic high molecular weight antioxidants, that is, catalase, superoxide dismutase, glutathione peroxidase, and glutathione reductase [20]. With the age, the malfunction of the corneal antioxidant defense mechanisms leads to ROS accumulation and the oxidative stress. The prooxidant/antioxidant imbalance is due to the inactivation of the antioxidant enzymes, mainly catalase, glutathione peroxidase, and lactate dehydrogenase, and leads to the functional and structural changes in the corneal tissue [21]. In response to the oxidative stress and with the age, through the apoptotic process the number of corneal fibroblasts and corneal endothelial cells declines in normal human corneas [22]. However, in specific conditions the increased ROS production and accumulation, the oxidative stress, and the prooxidant/antioxidant imbalance lead to the corneal pathologies.

The increased ROS levels and the oxidative stress both play a crucial role in the development of Fuchs endothelial corneal dystrophy, keratoconus, and granular corneal dystrophy type 2.

### 2.1. Fuchs Endothelial Corneal Dystrophy (FECD)

FECD is an oxidative stress disorder which leads to the age-related gradual loss of corneal endothelial cells (CECs), resulting in corneal edema and loss of vision. FECD affects approximately 4% of the population in the fourth or fifth decade of life and is characterized by an accelerated decrease of postmitotic endothelial cells density caused by apoptosis, as well as formation of posterior excrescences of Descemet's membrane, termed guttae, which arise as abnormal accumulation of subendothelial deposition of profibrotic extracellular matrix [23, 24]. Nondividing nature, positioned within the light path and high metabolic activity, predisposes CECs, mainly from the corneal center to the oxidative stress. Oxidative stress is the major contributor to the slow developing of CECs degeneration, loss of their hexagonal shape, and density in FECD patients [25]. FECD corneas exhibit increased accumulation of ROS (and RNS) in comparison with the normal tissues [21]. Central areas of FECD corneal buttons showed increased level of 8-hydroxy-2′-deoxyguanosine (8-OHdG), decreased numbers of mitochondria, and reduced activity of cytochrome oxidase (the major respiratory chain enzyme) in Jurkunas et al.'s study. According to the authors, increased level of oxidative mtDNA damage exhibited next to corneal guttae confirms association between macromolecular damage triggered by the oxidative stress and the endothelial cells apoptosis. Endothelial oxidative DNA damage is caused by imbalance of oxidant/antioxidant factors in CECs, based on an abnormal response of the transcription factor Nrf2 and its antioxidant targets, including superoxide dismutases [26]. Nrf2, nuclear factor-erythroid 2-related factor-2, is a key nuclear transcription factor coordinating upregulation of antioxidant defense in response to cellular stress. Nrf2 shows a high affinity to the antioxidant response element (ARE), which is involved in transcriptional activation of genes encoding proteins important for the protection against the oxidative stress, including peroxiredoxin, glutathione* S*-transferases, heme oxygenase-1 (HO-1), thioredoxin reductase 1 (TXNRD1), and ferritin [27]. Aberrant Nrf2 response influences the expression of multiple antioxidants in FECD corneal endothelium, causing accumulation of free radicals and other reactive species [23, 28]. Peroxiredoxins, which are involved in the removal of hydrogen peroxide from the cells and in the inhibition of ROS-induced apoptosis, show decreased expression in corneal endothelial cells and Descemet's membrane of FECD corneas [28]. Matthaei et al. showed decreased transcriptional levels of superoxide dismutase 1 and superoxide dismutase 2 in FECD endothelial samples and overexpression of NOX4 enzyme (NADPH oxidase 4) [29]. NAPDH oxidase, (NOX) family, is a reduced form of nicotinamide adenine dinucleotide phosphate and one of the most important sources for ROS generation [30]. Decreased levels of SOD1 and SOD2, decreased Nrf2-expression, and augmented NOX4 activity significantly exacerbate antioxidant/oxidant imbalance and contribute to consecutive induction senescence of CECs in FECD patients [29]. Lack of Nrf2 leads to perturbation of tissue homeostasis and activates p53-dependent apoptotic pathway in FECD endothelial cells [31]. On the other hand, experimental applying of sulfolane, which is Nrf2 agonist, caused cytoprotective effect by significant upregulation of major ARE-dependent antioxidants, decreased intracellularly ROS production, and ameliorated oxidative stress-induced CECs apoptosis in FECD corneas [32].

### 2.2. Keratoconus (KC)

KC is a degenerative disorder characterized by the corneal ectasia (stromal thinning leads to cone-shaped protrusion, often in the inferotemporal quadrant), associated with breaks in Bowman's layer and Fleischer's ring-iron deposits in the basal layer of the epithelium. Irregular astigmatism, myopia, and cornea scarring reduce the visual acuity. KC is mostly bilateral, with the onset usually in puberty and arresting around the fourth decade of life, affecting both genders and all ethnicities [33]. Visual impairment may be alleviated by spectacles or specialized contact lenses in most of the patients and in a part of them may be employed riboflavin-ultraviolet-A-induced collagen cross-linking therapy, which causes covalent bonding between the collagen fibrils and biomechanically strengthens the diseased cornea [34]; however, 10–20% of affected patients may necessitate the corneal transplantation [35]. Oxidizing UV radiation and blue light, genetic predispositions, and the environmental mechanical influences such as contact lens wear, atopy, and eye rubbing play a role in KC pathogenesis [36–38].

In comparison with the normal cornea samples, human KC corneas exhibit an increased stress-induced ROS generation, including superoxide [39], as well as the accumulation of nitrotyrosine, a marker for the formation of peroxynitrite, and increased production of nitric oxide (NO) radicals; elevated amounts of endothelial nitric oxide synthesis (eNOS) were detected at the site of Bowman's layer breaks [21]. Increased ROS and RNS formation lead to oxidative stress [37] and cause mtDNA impairment in keratoconus corneas.

Apparent and important role of mtDNA damage in the development of KC pathogenesis confirm many studies. In comparison with the age-matched normal corneas, keratoconus corneas present an increased level of mtDNA damage, which affects the protein-coding mtDNA region and disturbs the mitochondrial process of oxidative phosphorylation. Aberrations in the expression of oxidative phosphorylation proteins lead to improper ATP synthesis, increased ROS/RNS formation, and then in turn the further oxidative damage and increased ROS/RNS formation. KC corneas exhibit decreased activity of complex IV subunit 1 (CO I) in areas of corneal thinning [40]. KC stromal fibroblasts also show in vitro increased mitochondrial cytochrome oxidase subunit 2 (CO II) RNA levels in comparison with the normal cultures [41]. Han Chinese population exhibited a significant (*p* = 0.0002) decrease of leukocyte mtDNA copy number in KC patients compared to control subjects, which remained even when age, gender, and mtDNA genetic background were considered. Leukocytes are normal tissues in keratoconic corneas and leukocyte mtDNA copy number represents the general mtDNA copy numbers of the individual. There was no correlation between mtDNA haplogroup and the risk of keratoconus in this group [42], contrary to Saudi Arabian population, in which increased risk to develop KC in individuals correlated with the mitochondrial haplogroups H and R [43]. According to the authors, decreased leukocyte mtDNA copy number in keratoconus patients represents a genetic susceptibility to KC. Moreover, it is a predisposing factor for disease development by influencing oxidative stress, since decreased mtDNA copy number sustains imbalance between damaged and normal mtDNA, which favours further increasing ROS formation and additional oxidative stress in KC cornea [42].

KC corneas exhibit disturbance in the level of transcripts and/or activities of different antioxidant enzymes [44]. The activity of extracellular superoxide dismutase [45] and content of glutathione [37] are decreased in KC corneas in comparison to the normal samples.

Increased ROS formation, oxidative stress, and mtDNA damage and decreased antioxidant defenses in KC corneas cause keratocyte apoptosis and unfavourable changes in extracellular matrix (ECM), which finally lead to thinning and deformation of keratoconus corneas. Oxidative stress accelerates keratocyte apoptosis [41]. Integrity of mtDNA plays an important role in viability of cells and the mitochondrial dysfunction contributes to KC deformation [40].

Oxidative stress changes expression of two structural components of ECM, that is, collagen type XVIII/endostatin and collagen type XV. Remodeling of the extracellular matrix makes stroma more susceptible to degradation and results in its thinning [46]. Degradation of collagen components of the extracellular matrix in keratoconus corneas favours changes in the expression of ECM enzymatic regulators, that is, gelatinase A and matrix metalloproteinase 2 (MMP-2), which digest the main structural elements of the ECM, namely, collagen IV, collagen V, fibronectin, and laminin [47, 48], and MMP-2 inhibitor, that is, tissue inhibitor metalloproteinase 1 (TIMP-1). KC corneas exhibit increased activity of gelatinase A and decreased mRNA expression and protein levels of TIMP-1 [49]. ROS/nitric oxide pathway degrade TIMP-1 and increase MMP-2 activity [50]. TIMP-1 plays a role in the inhibition of apoptosis in a variety of cell types; therefore lower amount of this protein is associated with fragmentation of the epithelium and stroma thinning of KC corneas [51].

In some patients, their genetic predisposition to keratoconus, that is, polymorphisms in COL4A3 and COL4A4 genes, encoding components of type IV collagen, a major corneal structural protein [52], and/or mutation in the superoxide dismutase 1 gene [53] may accelerate the corneal changes. However, Stabuc-Silih et al. did not confirm correlations between mutation of SOD1 gene and keratoconus [54].

### 2.3. Granular Corneal Dystrophy Type 2 (GCD2)

GCD2 is an autosomal dominant disorder caused by point mutations (R124H) in transforming growth factor-*β*-induced gene-h3 (BIGH3) and is characterized by age-dependent progressive accumulation of transforming growth factor-*β*-induced protein (TGFBIp) deposits in the corneal epithelia and stroma, which interferes with corneal transparency [55].

Choi et al. confirmed that the oxidative stress is also involved in the pathogenesis of GCD2, since GCD2 primary cultured corneal fibroblasts demonstrate increased intracellular ROS and H_2_O_2_ generation and they are highly susceptible to the oxidative stress-induced cell's death in comparison with the normal primary cultured corneal fibroblasts [56]. Choi et al. showed in the other study that melatonin, which is involved in the control of various physiological functions and also has antioxidant and antiapoptotic properties, protected GCD2 corneal fibroblasts against the paraquat- (PQ-) induced oxidative stress, since it reduced intracellular levels of H_2_O_2_ and increased expression of Cu/Zn-superoxide dismutase and glutathione reductase in fibroblasts GCD2 corneas [57].

Increased ROS production and increased level of the oxidative stress play role in the etiology of the superficial punctate keratopathy [58] and impair the corneal wound healing [59, 60].

## 3. The Role of ROS and the Oxidative Stress in Glaucoma Pathogenesis

Glaucoma, an age-dependent disease being more common in the elderly population, is one of the leading causes of irreversible blindness [61]. Glaucoma is an optic neuropathy characterized by the progressive degeneration of retinal ganglion cells (RGCs), which die through an apoptotic process [62]. Increased intraocular pressure (IOP) is a consequence of abnormal high resistance to aqueous humor drainage via the trabecular meshwork, causing anterograde/retrograde axoplasmic flow impairment (the mechanical theory of glaucoma), and it is the leading risk factor for RGCs apoptosis in glaucoma [63]. However, several concomitant factors such as increased ROS production and oxidative retina damage and imbalance between prooxidative and antioxidant capacity have been postulated as the crucial factors in early retinal injury [64], together with the reduced perfusion pressure in the blood vessels (the vascular theory of glaucoma), which also significantly contribute to the glaucomatous neurodegeneration [65]. In the glaucoma pathogenesis are involved the trabecular meshwork in the anterior chamber of the eye; RGCs and their axons in the posterior eye segment; and the lateral geniculate nuclei and the visual cortex in the central nervous system [66–68].

### 3.1. The Influence of ROS and the Oxidative Stress on the Human Trabecular Meshwork

The human trabecular meshwork (TM) is the most sensitive tissue of the anterior chamber to the oxidative damage, since it is hidden in the sclerocorneal angle and not directly exposed to light and in consequence has fewer, than cornea and iris, antioxidant defence [69]. ROS induced by light change the oxidant/antioxidant balance in the aqueous humor. The oxidative stress stimulates enzymatic antioxidant defence systems and decreases the total antioxidant potential in aqueous humor; therefore, the level and activity of protecting superoxide dismutase and glutathione peroxidase decrease and such oxidant/antioxidant imbalance causes TM cells impairment [70]. Human TM cells are in contact with the relatively high concentrations of hydrogen peroxide and such exposure to H_2_O_2_ has no effect on outflow in normal eyes; however, it causes a 33% decrease in outflow in reduced glutathione-depleted eyes [71]. Patients with primary open-angle glaucoma (POAG) expose higher susceptibility to oxidative damage, since their total reactive antioxidant potential is reduced by 60–70%, although the activity of antioxidative enzymes is increased by the same amount [70], and they show the reduced levels of glutathione in plasma [72]. POAG patients display a genetic background rendering them susceptible to ROS-induced damage, since there is a more frequent deletion of the gene encoding for GSH S-transferase compared with the control individuals [66].

Increased hydrogen peroxide levels and the oxidative stress damage mainly structural and functional components of mtDNA in TM endothelial cells; however, damage of proteins and membrane lipids also occurs [73].

Increased level of 8-OH-dG derived from guanosine oxidation is an established biomarker of oxidative DNA damage. In Sorkhabi et al.'s study, both aqueous and serum 8-OH-dG levels were significantly higher in glaucoma patients than in the control group (4.61 ± 2.97 ng/mL versus 1.98 ± 0.70 ng/mL, *p* = 0.002, and 17.80 ± 8.06 ng/mL versus 13.63 ± 3.54 ng/mL, *p* = 0.046, resp.), and total antioxidant status determined in serum and in aqueous humor was significantly lower in glaucoma patients than in control group (0.55 ± 0.13 mmol/lit. versus 0.70 ± 0.14, *p* = 0.001, and 0.23 ± 0.13 mmol/lit. versus 0.34 ± 0.15, *p* = 0.001, resp.) [74]. Trabecular meshwork samples of patients with POAG, obtained during the filtration surgery, also exhibited increased level of 8-OH-dG [75]. More recently, the altered stability of mRNAs in human TM cells exposed to oxidative stress has been reported as well [76]. Mitochondrial dysfunction and oxidative mtDNA impairment of the human TM endothelial cells occur in POAG and pseudoexfoliative glaucoma, however not in other types of glaucoma [77], and are proportional to the clinical symptoms of the POAG, that is, intraocular pressure elevation and visual fields damage [75], and contribute to POAG progression [73].

Elevated ROS concentration influences TM activating nuclear factor-*κ*B (NF-*κ*B) pathway and causes oxidative/peroxynitrite stress [78].

NF-*κ*B is a family of transcription factors which play critical roles in inflammation, immunity, cell proliferation, differentiation, and survival. Increased ROS generation results in sustained NF-*κ*B activation, which in turn induces the expression of proinflammatory markers, including endothelial leukocyte adhesion molecule-1 (ELAM-1), interleukin- (IL-) 1*α*, IL-6, and IL-8 [79]. Short-term ROS/NF-*κ*B pathway activation contributes to the decrease of IOP [80]; however, its chronic stimulation exerts pathological effects on the TM and leads to glaucoma progression [81]. Enhanced level of superoxide anions induces overproduction of nitric oxide (NO), which in turn reacts with H_2_O_2_ and produces toxic metabolites, reactive peroxynitrite (ONOO^−^). Physiological levels of NO play an important role in controlling ocular vascular tone and the blood flow. NO is synthesized from l-arginine by a family of nitric oxide synthase (NOS) isozymes which includes neuronal (n)NOS, endothelial (e)NOS, and inducible (i)NOS. nNOS and eNOS are constitutive Ca^2+^ (calcium)/calmodulin-dependent enzymes and are tightly controlled by mechanisms regulating physiological intracellular Ca^2+^ levels, whereas iNOS is Ca^2+^-independent [82]. The human TM endothelium cells, involved in modulating the permeability of the endothelial barrier, express mainly eNOS isoform (and significantly lower amount of nNOS), which physiologically regulates aqueous outflow by maintaining endothelial cell function. The oxidative/peroxynitrate stress leads to eNOS deficit, which alters TM mobility and causes its contractile dysfunction [83]. Moreover, ROS/ONOO^−^ stress induces and sustains inflammation and proliferation of TM endothelial cells [64], as well as breakage of mtDNA and impairment of mitochondrial respiration, and the energy failure leads finally to the endothelial cells damage [64, 84].

**Figure 1:**
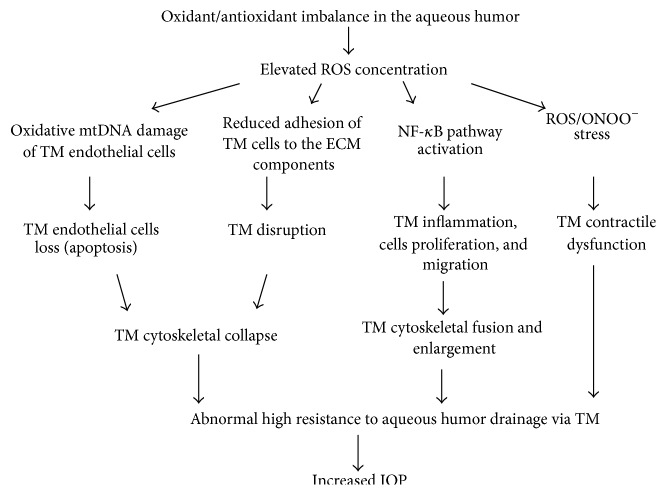
Schematic overview of the harmful influence of ROS and the oxidative stress on the trabecular meshwork structure and its function in glaucoma. ROS, reactive oxygen species; mtDNA, mitochondrial deoxyribonucleic acid; TM, trabecular meshwork; ECM, extracellular matrix; NF-*κ*B, nuclear factor-*κ*B; ROS/ONOO^−^, reactive oxygen species/peroxynitrite; IOP, intraocular pressure.

Increased hydrogen peroxide levels and the oxidative stress cause remodeling of TM cytoarchitecture and this leads to TM enlargement or collapse. The oxidative stress stimulates mobility (migration) of human TM cells in vitro, which causes trabecular thickening and fusion and contributes to trabecular enlargement [85]. Human TM cellularity declines linearly in relation to age; however, glaucomatous subjects have lower TM cellularity than nonglaucomatous subjects at the same age [86]. Human TM cells exposed to 1 mmol of H_2_O_2_ stress show reduced adhesiveness to the extracellular matrix structural components (to collagen types I and IV, laminin, and fibronectin) and such rearrangement of cytoskeletal structures may also lead to endothelial cells loss and cause TM disruption and collapse [87]. The exact mechanism of TM cells loss and the environmental factors contributing to it are not known yet; however, exogenous influence of higher H_2_O_2_ levels, combined with the insufficient glutathione level, may induce collagen matrix remodeling and cause trabecular cell apoptosis, independently of mitochondria [84]. TM plays a major role in the regulation of aqueous outflow [88]; therefore, its oxidative-related enlargement or collapse leads to the increased outflow resistance and elevation of IOP (Figure 1) [84].

### 3.2. The Influence of ROS and the Oxidative Stress on the Development of Glaucomatous Neurodegeneration

Increased IOP and/or hypoxia stimulate ROS production in glaucoma patients. Amplified ROS generation causes chronic stress state of the retina and of the optic nerve head tissue. ROS and the oxidative stress constitute an important noxious stimulus, which leads to progressive retinal ganglion cells (RGCs) loss in apoptotic and autophagic process, causes retinal and optic nerve glial dysfunction, and dysregulates ocular hemodynamics. In patients with glaucomatous neurodegeneration, progressive loss of RGCs and optic nerve axons leads finally to characteristic optic nerve atrophy and visual field defects.

#### 3.2.1. The Role of ROS and the Oxidative Stress in the Pathomechanism of RGCs Death

Oxidative mitochondrial dysfunction caused by amplified ROS generation (as well as nitric oxide-induced damage) is a part of the pathway for RGCs death and plays role in the development of glaucomatous neurodegeneration. Glaucomatous neurodegeneration means the impairment of RGCs (soma), as well as dendrites in the retina, axons in the optic nerve, and synapses in the brain. ROS may act as the direct cytotoxic stimulus causing RGC loss through apoptosis and as intracellular signaling molecules (second messenger parallel to neurotrophin deprivation) for RGCs death after axonal injury, responsible for transforming the information from the damaged axon into RGCs soma. ROS may be directly cytotoxic to RGCs in a caspase-dependent manner or may function in caspase-independent pathway. Caspase-independent apoptotic pathways can be activated by apoptosis-inducing factor. Extracellular ROS released from stressed cells into the extracellular milieu may facilitate RGCs degeneration additionally to the neurodegenerative injury induced by the intracellular ROS attack. Moreover, ROS released by the neighboring cells may also be cytotoxic to the primarily undamaged RGCs [12, 89].

In glaucoma patients, RGCs may also die in the course of dysregulated basal autophagic process [90] and ROS and the oxidative stress induce autophagy [91]. Autophagy refers to lysosomal degradation of cell's own constituents. This is highly conserved adaptive metabolic process, which permits the degradation and recycling of cellular constituents (including long-lived proteins and organelles), and is crucial for maintaining cellular homeostasis and cell survival under stressful conditions. Autophagy is triggered in response to various stressful conditions such as oxidative stress, hypoxia, ischemia/reperfusion injury, growth factor withdrawal, and nutrient deficiency. The autophagosome, a double membrane structure containing engulfed cytoplasm and its organelle content, fuses with lysosome(s) to create an autophagolysosome within which the endocytosed contents can be degraded by lysosomal enzymes. There are predominantly 3 autophagic pathways, that is, macroautophagy (autophagy), microautophagy, and chaperone-mediated autophagy (chaperones are a functionally related group of proteins assisting protein folding in the cell under physiological and stress conditions). Autophagy is primarily a nonselective degradation pathway but also different kinds of selective autophagy exist, that is, mitophagy, reticulophagy, pexophagy, xenophagy, and nucleophagy, which, respectively, refer to the selective removal of mitochondria, endoplasmic reticulum, peroxisomes, intruding microorganisms, and nuclei. Intralysosomal waste material accumulated with age and damaged cellular components, which are no longer functional, are also degraded by this self-eating process. If the autophagic degradative pathway is faulty, an accumulation of damaged proteins as aggregated deposits takes place which may cause the anatomical obstacles to physiological processes. Diminished autophagic activity plays a major role in aging and age-related diseases [92]. Mizushima subclassified autophagy into “baseline” and “induced.” Neuronal autophagy is protective at basal levels and prevents the aggregation of damaged organelles and the accumulation of proteins in neurons, thus promoting axonal homeostasis and efficient clearance of cellular soma. However, with the progression of autophagy, neurons undergo stimulation, which deregulates the basal autophagic mechanism and converts it to induced autophagy [93].

Superoxide and hydrogen peroxide play a dual role signaling autophagy process. Through influence on BECN1 (Beclin 1, autophagy-related) ROS activate formation of class III phosphatidylinositol 3-kinase (PtdIns3K) complexes and positively regulate autophagy; PtdIns3K is an intracellular energy sensor, which specifically responds to the energy depletion [94]. BECN1 contributes to the early formation of autophagic vesicles [95] and is predominantly located in RGCs layer of the retina [96]. However, ROS also can activate BCL2 (B-cell CLL/lymphoma 2) family proteins, which after binding to BECN1 disorganize the formation of PtdIns3K and disrupt the induction of autophagy [94].

Both chronic hypertensive glaucoma and/or conditions of retinal transient ischemia, induced by acutely increasing IOP, stimulate ROS production and dysregulate basal autophagy. Autophagy is originally activated in the dendrites of RGCs to promote cellular protection and maintain intracellular homeostasis. Axons contain a large number of mitochondria, which make them more sensitive to chronic ischemia. Neuroprotective effect of autophagy in axons lessens after 4 weeks of progressive increase of IOP. Thereafter, autophagy is predominantly activated in the neuronal soma, which disrupts homeostasis, decreases cell viability, and triggers autophagic RGC death [90, 96]. If the IOP increases acutely, autophagy is enhanced immediately and occurs mostly in cell bodies, which induces neuronal cell death in a relatively short time [90].

#### 3.2.2. The Influence of ROS and the Oxidative Stress on Glial Cells of the Retina and Optic Nerve Head

Under oxidative conditions glial cell lessens its neuroprotective ability and may even become neurodestructive. ROS and the oxidative stress cause supportive glial dysfunction, which lead to the secondary RGCs apoptosis. ROS stimulate pathological glial activity (increase secretion of TNF-*α* and nitric oxide), cause oxidation of its protein (glutamine synthetase), and damage glial cells through activation of AGE/RAGE signaling pathway. ROS induce glial immune system as well, which additionally facilitate the progression of glaucomatous neurodegeneration [12].

ROS and aging promote accumulation of advanced glycation end products (AGEs), a hallmark of many chronic neurodegenerative diseases. AGEs stimulate ROS production and assist development of glaucomatous neurodegeneration [12]. AGEs are proteins, lipids, or nucleic acids formed by nonenzymatic glycation or glycoxidated, after the exposure to the aldose sugars. The oxidative stress increases with age and, furthermore, the ability to respond to the oxidative stress declines with age, mostly due to the imbalance between increasing oxidant production and decreasing antioxidant capacity and AGEs accumulate in various tissues in the course of physiological aging. Due to their synergism with the oxidative stress, AGEs production is promoted by the oxidative stress; while AGEs lead to ROS generation, AGEs are commonly implicated as factor which exacerbates progression of many neurodegenerative diseases. These detergent insoluble and protease-resistant, nondegradable AGEs aggregates may impair normal cellular/tissue functions directly, or indirectly, by AGE/RAGE pathway after binding to the specific receptors for advanced glycation end products (RAGEs). AGE/RAGE pathway results in the cell activation, proliferation, apoptotic cell death, chemotaxis, angiogenesis, and ROS generation. As a consequence of AGEs accumulation, many proteins lose their function, including proteins involved in the regulation of gene transcription [97].

In glaucoma, AGEs are accumulated predominantly extracellularly in laminar cribriform plates and blood vessels of the optic nerve head, mainly on many long-lived macromolecules like collagen [98]. Extracellular aggregates increase tissue rigidity and mechanical strength and increase impairment of microcirculation, which additionally facilitate injury of axons damaged by increased IOP [12]. AGEs are also accumulated intracellularly in RGCs, glial cells, and axons as well [98], which cause intracellular protein traffic and impair axonal transport [12]. Receptors for AGEs are upregulated predominantly on Müller cells of retinal glial cells and on RGCs [98]. It makes glial cells and RGCs particularly susceptible to AGE/RAGE signaling pathway and such receptor-mediated signaling may amplify direct cytotoxic effect caused by extracellular/intracellular AGEs accumulation [12].

The retina and optic nerve glial cells vigorously respond to ROS stimulation. Activated autoimmune response may facilitate primary and/or secondary RGCs degeneration through stimulation of an aberrant immune response [12]. In Tezel et al.'s study, glial cells exposed to ROS upregulated major histocompatibility complex (MHC) class II molecules, important in autoimmune response. T cells recognize antigens in the form of small peptides tightly bound to MHC class II molecules displayed on the surfaces of antigen-presenting cells and MHC complex interacts with antigen-specific receptors on T cells to induce an antigen-specific reaction. Compared with the control, glial cells in ROS-generating systems were more potent inducers of T cell activation in a cell density- and time-dependent manner, assessed by increased T cell proliferation (approximately threefold) and TNF-*α* secretion (approximately sixfold), *p* < 0.01 [99].

McElnea et al. confirmed the influence of ROS overproduction, oxidative stress, and mitochondrial dysfunction (as well as impaired calcium extrusions) on glial cells of the optic nerve head in glaucomatous patients. The compare levels of the oxidative stress, mitochondrial function (as well as calcium homeostasis) in glial fibrillary acid-negative protein lamina cribrosa cells obtained from the optic nerve head region of glaucomatous lamina cribrosa (GLC), and normal lamina cribrosa (NLC) human donor eyes showed that intracellular ROS production was increased in GLC compared to NLC (27.19 ± 7.05 *μ*M MDA versus 14.59 ± 0.82 *μ*M MDA, *p* < 0.05); malondialdehyde (MDA) is a naturally occurring product of lipid peroxidation used as an indicator of the oxidative stress. Moreover, mitochondrial membrane potential was lower in GLC (57.5 ± 6.8%) compared to NLC (41.8 ± 5.3%), expression of the antioxidants (aldo-keto reductase family 1 member C1 and glutamate cysteine ligase catalytic subunit) was significantly (*p* = 0.02) lower in GLC compared to NLC control, and intracellular calcium (Ca^2+^) levels were significantly higher (*p* < 0.001) in GLC cells compared to NLC [100].

ROS and the oxidative stress have influence on the optic nerve tissue remodeling, since they trigger neuronal loss, disorganize laminar cribriform plates secondary to extracellular AGEs accumulation, and stimulate matrix metalloproteinases (MMPs) for digestion of extracellular matrix (ECM) in glaucomatous eyes. Degradation of the optic nerve neurons progresses and optic disc cupping enlarges despite glial activation and increased glial production of the extracellular matrix molecules [12].

#### 3.2.3. The Influence of ROS and the Oxidative Stress on Ocular Hemodynamics

**Figure 2:**
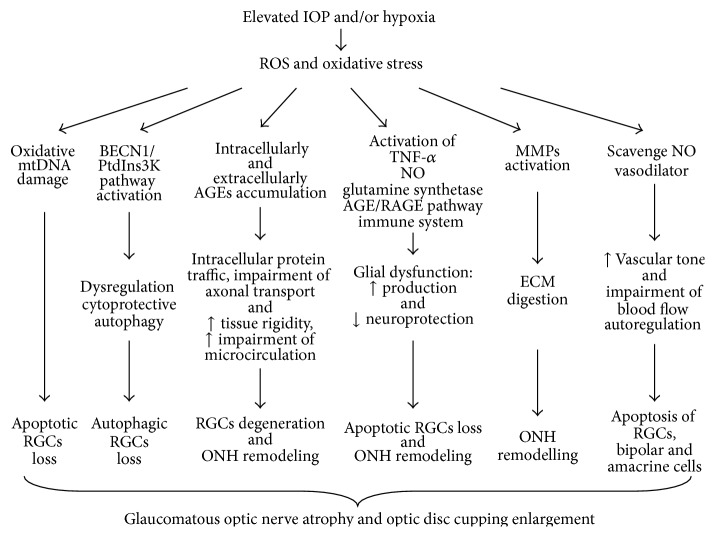
Schematic overview of the influence of ROS and the oxidative stress on the retina and the optic nerve head changes in the course of glaucomatous neurodegeneration. IOP, intraocular pressure; ROS, reactive oxygen species; mtDNA, mitochondrial deoxyribonucleic acid; RGCs, retinal ganglion cells; BECN1/PtdIns3K, Beclin 1/phosphatidylinositol 3-kinase; AGEs, advanced glycation end products; ONH, optic nerve head; TNF-*α*, tumor necrosis factor alpha; NO, nitric oxide; AGE/RAGE, advanced glycation end product/receptor for advanced glycation end product; MMPs, matrix metalloproteinases; ECM, extracellular matrix.

ROS and the oxidative stress lead to dysregulation of ocular hemodynamics, which also contribute to RGCs apoptosis. In the eye, the nature of disturbed hemodynamics is characterized as an altered regulation of perfusion in the terminal or preterminal arterial vasculature, however not as an obstructive vascular disease like arteriosclerosis [101]. ROS scavenge NO radicals and thus remove an important vasodilator resulting in vasoconstriction. NO is a potent signaling molecule in the blood vessels, where its continuous formation from endothelial cells acts on the underlying smooth muscle to maintain vasodilatation and blood flow and plays an important role in controlling ocular vascular tone and blood flow in the human eye [102]. ROS increase vascular tone and impair autoregulation of blood flow. In Zeitz et al.'s study, ROS induced in vitro a transient increase of vascular tone (transient contractions) of isolated rings of porcine posterior ciliary arteries. The shape of these contractions had parallels with vasospasms. Short-time exposure alters vascular tone which was totally reversible and the maximal force generation potential was unchanged; however, the arterial ring preparation lost its excitability after the prolonged ROS exposure [103]. Glaucoma progression is associated with the decreased blood flow velocities in the short posterior ciliary artery [104]. Excessive elevation of the intraocular pressure leads to retinal ischemia-reperfusion (I/R) insult. This strong prooxidant condition stimulates ROS production and predisposes retina to the oxidative damage. In animal models, transient acute I/R injury and impair blood flow dynamics resulted in necrosis and apoptosis of cells in both the ganglion cell layer and inner nuclear layer [105]. The schematic overview of the role of ROS in the development of glaucomatous neurodegeneration is presented in Figure 2.

## 4. The Role of ROS and Oxidative Stress in Leber's Hereditary Optic Neuropathy and in the Traumatic Optic Neuropathy

### 4.1. Leber's Hereditary Optic Neuropathy (LHON)

LHON is an acute or subacute bilateral central vision loss, due to optic nerve degeneration, and occurring predominantly in young males. LHON is the most frequent mitochondrial disease, due to mtDNA point mutations (positions 11778, 3460, and 14484) coding for proteins in mitochondrial electron transport chain complexes I and III. mtDNA mutations lead to loss of axons and their RGCs soma in apoptosis process [106, 107]. The primary cause of the disease is clearly known; however, the mechanism of relatively selective loss of the smaller RGCs and their axons remains enigmatic, and enhanced H_2_O_2_ production by the mutant mitochondrial complexes has been hypothesized as etiological factor [107], since (i) mitochondrial complexes I and III are the main sources of basal superoxide production and aberrant production of H_2_O_2_ from mutated METC components may cause RGCs death [106], (ii) RGCs use superoxide as an intracellular signal for initiating the apoptosis [108], probably by oxidizing critical sulfhydryls in signaling macromolecules [109], and (iii) RGCs death is triggered when the mitochondrial superoxide levels are increased by knocking down mitochondrial superoxide dismutase 2 [110].

The effect of LHON mutations has been studied by producing transmitochondrial cybrids in human cell lines, which demonstrate upregulation of some mtDNA transcripts and exhibit increased superoxide production compared to wild-type cells. In Giordano et al.'s study, LHON cybrids presented overproduction of ROS, as well as decrease in mitochondrial membrane potential, increased apoptotic rate, loss of cell viability, and hyperfragmented mitochondrial morphology compared with control cybrids [111]. In another study, LHON cybrids carrying the np11778 mutation became selectively more H_2_O_2_ sensitive compared with the parental cell line. They contained a decreased cellular glutathione pool (49%, *p* ≤ 0.05), despite 1.5-fold enhanced expression of the regulatory subunit of *γ*-glutamylcysteine synthetase (*p* ≤ 0.05). The capacity to detoxify H_2_O_2_ was reduced although the activity of superoxide dismutase, glutathione peroxidase, and glutathione reductase was unchanged [107].

### 4.2. ROS Overproduction Contributes to the Traumatic Optic Neuropathy (TON) Pathogenesis

TON means partial or complete loss of optic nerve function due to the direct or indirect optic nerve injury, after head trauma sequelae such as edema, hemorrhage, and concussion [112]. RGCs apoptosis after optic nerve injury is caused by lack of neurotrophin support, increased extracellular glutamate levels, disruption of cellular homeostasis, and damage from free radicals. Apoptotic processes are also activated by microglial cells, which release inflammatory mediators (cytokines, prostaglandins, and complement molecules) and reactive oxygen species [113]. Ahmad et al. confirmed the increased oxidative stress in mice retina with TON [114]; however, treatment with ABT702 (pharmacological adenosine kinase inhibitor) attenuated neurotoxicity and significantly decreased levels of the oxidative stress markers, that is, superoxide anion, iNOS/nNOS, and nitrotyrosine, and attenuated inflammation (decreased expression of many inflammatory molecules mediated by adenosine) in retinal sections of mouse with TON [115]. ROS are also overproduced during secondary degeneration following neurotrauma, which means that the precise impairment of only dorsal axons of optic nerve causes secondary degeneration of intact ventral axons [116]. In experimental study, the level of ROS (and nitrogen species) increased at 1, 3, and 7 days in ventral optic nerve after dorsal injury. Immunoreactivity for glutathione peroxidase and heme oxygenase-1 increased in ventral optic nerve at 3 and 7 days after injury, respectively. Despite the increased antioxidant immunoreactivity, DNA oxidation was evident just since the 1st day, lipid oxidation after 3 days, and protein nitration after 7 days since the injury. Oxidative (and nitrosative) damage was particularly evident in CC1-positive oligodendrocytes [117].

## 5. The Role of ROS and the Oxidative Stress in Cataractogenesis

Cataract, the opacification of the crystalline lens, is one of the leading causes of blindness in the world and aging is the greatest risk factor for noncongenital cataract formation. However, it is a multifactorial optic disorder and other factors like exposure to sunlight UV radiation, smoking, diabetes, malnutrition, myopia, and drug (steroid) use also contribute significantly to cataractogenesis [118].

The progressive loss of lens transparency associated with the increasing age is a cumulative physiological response to toxic environmental factors leading to an excessive generation of ROS in the lens epithelium cells (LECs) and in the superficial lens fiber cells, as well as in the aqueous humor [119]. UV-induced oxidative damage is a significant contributory factor to cataractogenesis. The eyes are continuously exposed to solar radiation, which can be divided into five regions in increasing order of wavelengths, that is, ultraviolet UVC, UVB, UVA, visible range, and infrared range. Among these radiations, UVC and UVB are responsible for photochemical reactions. UVA, visible range, and infrared range radiation are traditionally thought to be less damaging [120]. The major effect of UV radiation is through photochemical generation of ROS, including superoxide and its derivatization to other potent entities such as hydrogen peroxide, hydroxyl radicals, and singlet oxygen in the lens and in aqueous humor, which lead to oxidative damage of the lens tissue [120, 121]. The incidence of cataract is higher in the population which is more exposed to the sunlight [122].

Under normal conditions the lens is well equipped and uses multiple physiologic defense strategies to scavenge ROS and to maintain them at low levels to protect the lens from the toxic effects of oxidative damage. The lens defense system constitutes enzymatic antioxidants, that is, superoxide dismutase (SOD), catalase (CAT), and glutathione peroxidase (GPX) utilizing H_2_O_2_, chemical antioxidants, that is, alfa-tocopherol, beta-carotene, ascorbate, and GSH, structural antioxidants, that is, cholesterol and membrane protein, and transition metal-sequestering protein including aqueous and plasma ceruloplasmin [123, 124]. Enzymatic defense system also protects the lens from lipid-derived hydrogen peroxide [123]. However, protective systems decrease with the age and long-term exposure to oxidative stress predispose lens cells at risk for cumulative oxidative damage and cataract formation [118]. The effectiveness of SOD and other antioxidant enzymes is limited to several reasons such as the following: (i) their deactivation with aging and their selective distribution and availability in various cellular compartments, (ii) being macromolecules, where they cannot penetrate the certain sensitive sites of oxidation in nucleic acids and in proteins, (iii) the fact that the lens is surrounded and bathed by aqueous and the vitreous humors, fluids which lack the enzymatic defenses. Therefore the lens cell membranes, which are continuously exposed to a photochemical oxidative environment due to the continued light penetration during the long periods of photopic vision, remain susceptible to photo damage [121]. Human mature cataractous lenses show decreased activity of SOD, glutathione peroxidase (reducing organic hydrogen peroxide, including hydrogen peroxide of lipids) and glutathione reductase, and however no signs of deficiency in activities of catalase [123]. Genetic variations in the antioxidant genes coding for the SOD, CAT, and GPX enzymes may also lead to decreased or impaired regulation of their enzymatic activity and alter ROS detoxification. Zhang et al.'s study showed that the G/G genotype of the SOD1-251A/G polymorphism may be associated with an increased risk of cataract. However, in CAT-21A/T and GPX1-198C/T polymorphisms, there were no significant differences in the variant homozygous frequencies in age-related cataract patients in comparison to controls [125].

### 5.1. The Role of ROS in Oxidative Stress-Induced Mitochondria-Dependent Apoptosis in Human Lens Epithelial Cells

Oxidative stress of the lens is not only the result of an imbalance between lens oxidants and antioxidants but also the consequence of cellular redox status imbalance in the lens [123]. The short range packing of the crystallins, which make up over 90% of the soluble lens, must exist in a homogenous state, that is, in the redox balance, to maintain lens transparency, which means the necessity to maintain continuously the thiol (sulfhydryl) groups of proteins of the lens center in a nearly 100% reduced state to prevent formation of high molecular weight protein aggregates, which contributes to the cataract formation [118]. LECs are the center of lens metabolic activity and their oxidative damage plays a significant role in cataractogenesis [126]. Mitochondria are abundant in the lens but only within the epithelium and differentiating fibers; mature fibers in the core of the lens lack mitochondria [127]. Mitochondria of LECs and superficial fiber cells consume 90% of the oxygen entering the lens and are major endogenous sources of ROS [118, 128]. It is estimated that up to 1% to 5% of oxygen consumed by the lens mitochondria is converted to ROS [129].

Age-related mitochondrial dysfunction and ROS imbalance induce oxidative damage of cellular components and play crucial role in the pathogenesis of senile cataract development [118]. ROS are formed in LECs mitochondria as a byproduct of normal metabolism and as a consequence of exposure to environmental compounds and if not eliminated cause oxidative damage of DNA, proteins, and lipids [130].

Nucleic acids are prone to oxidative damage by ROS. Continuous attack of ROS leads to DNA oxidation. OH^•^ modify guanine of DNA and form 8-hydroxyguanine. 8-OHdG is highly mutagenic, causes GC to TA transversions, and has been commonly quantified as a steady-state estimate of oxidative stress in tissues [131]. The extent of lens DNA damage caused by direct ROS attack can be assessed by 8-hydroxyguanine assay and comet assay. Cultured normal human LECs show increase of 8-hydroxyguanine marker in response to the oxidative stress [132]. Quantitative assessment of DNA damage in LECs achieved from senile cataract patients and performed in comet assay showed smearing of DNA fragments instead of bands in the tail, which indicate random (nonenzymatic) damage with ROS, which act by chemical reaction [133]. Oxidative mtDNA damage is a causative factor in aging and a wide variety of degenerative diseases. mtDNA damage is more extensive and persists longer than nDNA damage because of its close proximity to ROS generation through the respiratory chain and its paucity of protective histones. A vicious cycle of mtDNA damage and ROS production established within cells leads to loss of the mitochondrial membrane potential and release of cytochrome c, resulting in the cell apoptosis [130]. Oxidative stress can disrupt the balance between ROS production and the radical scavenging effect and lead to apoptotic cell death through the mitochondrial apoptosis pathway. Numerous studies of human cataractous lenses confirmed extensive oxidative damage of mtDNA and membrane pumps of lens cells, as well as increased unscheduled expression of genes stimulated by excessive ROS production. mtDNA damage and pathological gene expression are both responsible for loss of LECs viability and their death by apoptotic and necrotic mechanisms. LECs death by apoptosis plays key role in the pathogenesis of noncongenital cataract development in human [118, 134–136].

Increased accumulation of oxidized proteins, mainly methionines and cysteine residues, also is linked to cataractogenesis and confirms age-related increase in rates of ROS generation, decrease in antioxidant activity, and loss in the capacity to degrade oxidized proteins [137]. Moreover, excessive ROS production and oxidative stress lead to formation of lipid peroxides, which contribute to pathological processes of aging and play role in systemic (diabetes, atherosclerosis, chronic renal failure, and inflammation) and retinal degenerative diseases, and they are statistically significant risk factors for cataract development [123]. Lipid peroxides impair both cell membrane and cytosol regions [138], damage DNA [139], induce a drop in total glutathione and dramatic change in the redox ratio of glutathione, and lead to the appearance of new fluorophores and large protein aggregates with low solubility (clouding matrix) in the lens matter [140].

## 6. The Role of ROS and the Oxidative Stress in Diabetic Retinopathy

Diabetes mellitus (DM) is a chronic and progressive neurodegenerative disease, characterized by chronic hyperglycemia and altered cellular homeostasis, which lead to the diffuse microvascular and macrovascular damage, numerous complications, and multiorgan dysfunction. During the course of DM, every cell is exposed to the abnormally high glucose concentrations; however, high glucose-related damage only targets specific tissues, that is, retina, nerve tissues, and kidney, since these tissues are deficient in the ability to change glucose transport rates when faced with the elevated extracellular glucose concentrations [141].

Diabetic retinopathy (DR) is a chronic and progressive complication in the course of diabetes mellitus type 1 or type 2 and the major cause of blindness in people of working age. It develops over approximately 10 to 25 years, and during the first two decades of the disease, nearly all individuals with type 1 and approximately 60% of individuals with type 2 diabetes will have some degree of retinopathy [142]. Diabetic retinopathy is one of the microvascular diabetes complications, characterized by gradual and progressive alterations in the retinal microvasculature with accompanying damage of glia and neurons [143]. DR results from capillaries damage. Capillaries are lined with endothelial cells, surrounded by smooth muscle cells and sealed by pericytes, which provide tone to the vessels and create a blood barrier for closed capillaries in the retina and in the choroid. In early DR stage, pericyte and endothelial cells undergo accelerated death by apoptosis, which leads to the reduction of pericyte numbers manifested by their degeneration (ghost cells) or loss, followed by the increased numbers of acellular-occluded capillaries, microaneurysms, and capillary basement membrane thickening. Noncapillary cells (Müller cells and other glial cells) are also lost selectively via apoptosis. Unsealed capillaries begin to leak plasma and erythrocytes into the surrounding retinal tissue, resulting in edema and intraretinal hemorrhages. Endothelial cells try to repair the damage by multiplying on the inner membrane; however, it leads to the capillaries occlusion and ischemic retina releases the growth factors leading to the pathological angiogenesis [144]. Based on the extent of microvascular damage, DR is classified into either nonproliferative (mild, moderate, or preproliferative, characterized by cotton wool spots, venous beading and loops, blood vessel closure, tissue ischaemia, and the formation of intraretinal microvascular abnormalities) or proliferative. In proliferative DR, pathological angiogenesis is driven by the vascular endothelial growth factor (VEGF) sourced from retinal vascular pericytes, retinal ganglion cells, and glia. Vision loss occurs from breakdown of the blood-retinal barrier, resulting in macular edema, inner retinal and vitreous hemorrhages, and tractional retinal detachment [144].

### 6.1. The Interaction between Hyperglycemia, ROS Stress, and Hyperglycemia-Induced Metabolite Pathways

Hyperglycemia stimulates overproduction of mitochondrial ROS and generates the oxidative stress. Increased mitochondrial ROS levels activate the poly-ADP-ribose polymerase (PARP) pathway, which reduces glyceraldehydes 3-phosphate dehydrogenase (GAPDH) activity. Decreased GAPDH level in turn contributes to overactivation of four classic hyperglycemia-induced metabolite mechanisms, that is, the polyol pathway, the protein kinase C (PKC) pathway, AGEs pathway, and the hexosamine pathway. The reduction of GADPH activity can be prevented by MnSOD [145–147].

According to Brownlee, all four classic hyperglycemia-induced pathways are activated by a single upstream event; that is, the mitochondrial overproduction of ROS and all four pathways become the source of increased ROS production and stimulation of the oxidative stress [145, 146]. All classic hyperglycemia-induced metabolite mechanisms result in decreased NADPH levels and increased NADPH oxidase (Nox) levels (see below). NADPH regenerate glutathione, an important scavenger of ROS. Therefore, decreased levels of NADPH are responsible for the increased ROS accumulation and the oxidative stress damage. Moreover, decreased NADPH levels lead to the inhibition of GAPDH, which in turn activate four hyperglycemia-induced pathways [145, 146]. The unifying mechanism proposed by Brownlee interconnects increased ROS production and the oxidative stress with four main hyperglycemia-induced processes and explains the hyperglycemia-induced endothelial cells damage (apoptosis) and the progression of diabetic retinopathy [145].

ROS and the oxidative stress contribute to “metabolic memory” or “legacy effect.” It means that the diabetic retinopathy progress, even after glycemia, has been normalized and well controlled. Mitochondrial abnormalities are irreversible, even after hyperglycemia stress is terminated and these impaired mitochondria are source of permanent ROS overproduction [145]. In laboratory conditions, human ARPE-19 retinal cells [148] and retinal pericytes [145] continue ROS overproduction even after the glucose normalization. Hyperglycemia, increased ROS production, the oxidative stress, and excessive AGE formation are causally associated with and show linear relationship in the early years of diabetes. ROS, which act at the mitochondrial level, are associated with “micro” metabolic memory and form the lowest denominator of diabetic complications. However, persistent mtDNA damage and respiratory chain protein glycation generate AGEs, which stimulate ROS production, and more ROS amplified AGEs formation and such vicious cycle acts independently of hyperglycemia level in advanced diabetes stage. AGEs, which are result of chronic interaction with the oxidative stress at tissue/vessel level, are associated with “macro” metabolic memory and form the bridge between micro- and macrovascular diabetic damage [149].

Intracellular AGEs formed via nonenzymatic glycation and glycoxidation processes [146] on short half-life proteins lead to endothelial cells dysfunction, loss of pericytes, and neuronal cells damage. They also reduce platelet survival, increase platelet aggregation, promote a procoagulant state, lead to ischemia, and induce growth factors, which stimulate pathological angiogenesis [150]. AGEs formed on long half-life proteins like collagen modify extracellular matrix and cause loss of charge and structural distortions associated with a lower integrin binding affinity, which leads to cell detachment, basement membrane thickening, and their resistance to proteolytic digestion [151]. AGEs/RAGE pathway increases cytosolic ROS level, activates NF-*κ*B pathway, and increases expression of cytokine and adhesion molecule. RAGEs activate indirectly the toll-like receptor 4 (TLR-4), which can trigger interaction with an innate immune system as well in type 2 diabetic patients [152].

### 6.2. The Influence of ROS and the Oxidative Stress on Endothelial Cells Apoptosis in Diabetic Retinopathy

Sustained hyperglycemia and increased chronic local oxidative stress disrupt retinal metabolism and accelerate premature endothelial cells apoptosis via mitochondrial dysfunction in both type 1 and type 2 diabetes retinopathy. In the early stages of diabetes, increased mtDNA biogenesis and repair compensate the ROS-induced damage. However, while it sustained insulting, this mechanism is overwhelmed and both the function and structure of mtDNA are damaged (mitochondrial electron transport chain is highly sensitive to the oxidative stress) [153–155]. The compromised electron transport chain propagates a vicious cycle of ROS and the dysfunctional mitochondria fuel loss of capillary endothelial cells by initiating their apoptosis [153–155]. Poor glycemia control and chronically increased intracellular glucose flux decrease retinal mtDNA copy number [156]. In diabetic retinopathy, the mtDNA damage at the regulatory region (the displacement loop) is considerably higher in comparison to other mtDNA portions [156]. The enzymes important for mtDNA repair, that is, 8-oxoguanine DNA glycosylase (OGG1), MutY homolog, and thymine DNA glycosylase, become subnormal, and the transcription and replication mechanisms including mitochondrial transcription factor A (TFAM) and polymerase gamma (POLG) are also compromised [156, 157].

In diabetic retinopathy, increased ROS expression and the oxidative stress may induce endothelial cells senescence via downregulation of Sirt6. Sirt6 is a nuclear chromatin-bound protein, which regulates glucose homeostasis [158], has antiaging and anti-inflammatory properties, and is involved in the oxidative stress-induced endothelial cells senescence pathomechanism [159, 160]. In Liu et al.'s study, Sirt6 protein was markedly reduced in endothelial cells activated by H_2_O_2_, and overexpression of Sirt6 partially reversed H_2_O_2_-induced endothelial cells dysfunction and senescence symptoms like decrease in endothelial cells growth, proliferation and angiogenic ability, loss of eNOS protein, and increase in senescence markers. According to the authors, induced by the oxidative stress, downregulation of Sirt6 may be involved in the pathogenesis of diabetic retinopathy [161].

### 6.3. The Influence of ROS on Retinal Neuronal Cells Apoptosis in Diabetic Retinopathy

ROS not only influence retinal vasculature but also exert neurodegenerative impact on diabetic retina [162, 163], which confirm results of the experimental studies.

In the streptozotocin- (STZ-) induced type 1 diabetes model mouse, cross-talk between ROS and renin-angiotensin system led to the reduction level of synaptophysin (synaptic vesicle protein for neurotransmitter release), most likely through excessive protein degradation by the ubiquitin-proteasome system. Moreover, ROS also decreased brain-derived neurotrophic factor (BDNF), which regulates axonal growth, synaptic activity, and neuronal survival. The damage of synaptic transmitter and degradation of neurotrophic factor, stimulated by the excessive ROS level, caused neuronal cells apoptosis and visual impairment [162]. However, constant lutein (antioxidant) treatment of the STZ-induced diabetes model mice (which presented synaptophysin and BDNF reduction caused by H_2_O_2_ stimulation) suppressed decreasing of synaptophysin protein and electroretinography impairment and preserved neuronal cells survival [163]. Lutein is a yellow pigment, which filters the high-energy blue light being toxic to the retina. The preventive effect of lutein observed in the mice diabetes model occurred by lutein antioxidative influence and the ROS reduction and not because of the filtering light of high energy [164].

Experimental studies show that AGEs also affect adversely the whole diabetic neurosensory retina. Intracellular AGEs accelerate directly neuronal cells apoptosis and extracellular AGEs (which alter metabolism of neuroretinal supporting cells) accelerate indirectly neuronal apoptosis [165]. Müller cells play fundamental role in retinal physiology. However, macroglia stimulated by hyperglycemia increases ROS production and amplifies AGEs formation and becomes dysfunctional due to increase glial fibrillary acidic protein (GFAP) expression, NO production, and glutamate synthesis (as a function of glutamate transporter disruption), and in consequence Müller cells contribute indirectly with retinal neurons excitotoxicity to the diabetic retina [166]. In connection with above Chilelli et al. suggest that diabetic retinopathy can be a sensory neuropathy, like peripheral diabetic neuropathy [149].

### 6.4. The Role of ROS in Stimulating Local Inflammation and Pathological Angiogenesis in Diabetic Retinopathy

The oxidative stress and inflammatory processes play the important roles in the development of microvascular lesions characteristic for diabetic retinopathy. The oxidative stress regulates expression of proinflammatory proteins [167, 168]. The oxidative stress and inflammation promote endothelial cells senescence [169] and pathological angiogenesis characteristic for proliferative diabetic retinopathy [170].

Reactive oxygen species in the retina may stimulate retinal angiogenesis by many molecular pathomechanisms. ROS participate in the activation of proinflammatory NF-*κ*B pathway, which in turn leads to the production of tumor necrosis factor alpha (TNF-*α*) and subsequent generation of inflammatory and angiogenic mediators such as interleukin 6 (IL-6), interleukin 8 (IL-8), cyclooxygenase 2 (COX-2), intercellular adhesion molecule 1 (ICAM-1), monocyte chemoattractant protein 1 (MCP-1), and VEGF [170].

Mitochondrial derived ROS trigger pathological angiogenesis by stabilization HIF-1*α* factor. Hypoxia-inducible factor-1 (HIF-1), the main regulator of oxygen homeostasis, consists of HIF-1*α* and HIF-1*β* subunits. Under hypoxic conditions, HIF-1 activates the transcription of a broad variety of genes, including those encoding erythropoietin, glucose transporters, glycolytic enzymes, inducible nitric oxide synthase, heme oxygenase-1, VEGF, and others, to ensure cell survival under conditions of hypoxic stress and to restore O_2_ homeostasis [171, 172]. Under normoxic conditions, HIF-1*α* is conserved by HIF prolyl hydroxylases (PHDs), which allows them to be rapidly degraded. However, under hypoxic conditions PHD is inhibited, since it requires oxygen for functioning and this results in the stabilization of HIF-1*α*. The stabilization of HIF-1*α* leads to the upregulation of many hypoxic-sensitive genes such as angiopoietin, erythropoietin, VEGF, and stromal cell derived factor-1 (SCDF-1). All of them exhibit angiogenic properties in the retina, resulting in pathological angiogenesis and vascular leakage [170, 171]. ROS can directly be ligated to the active ferrous iron center of PHDs and promote phosphorylation-dependent stabilization of HIF-1*α* [170, 172], which trigger pathological angiogenesis. Moreover, the relationships between the NF-*κ*B and HIF-1 pathways result in the amplification of signals of both pathways [170, 173].

**Figure 3:**
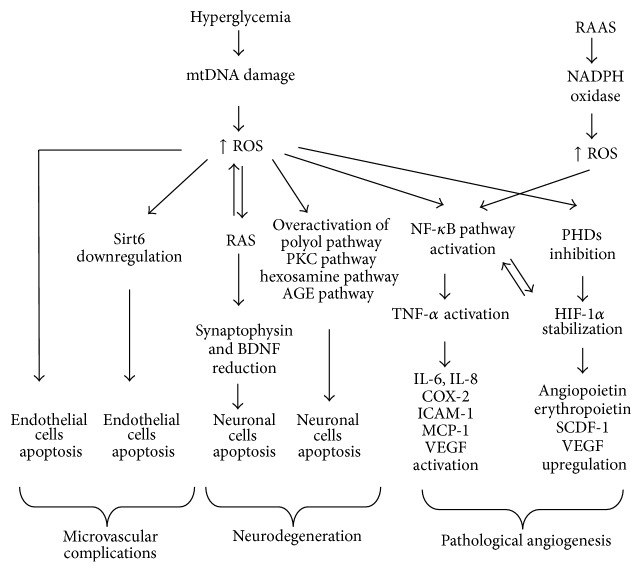
Schematic overview of the ROS influence on the development of microvascular complications, neurodegeneration, and pathological angiogenesis in the course of diabetic retinopathy. mtDNA, mitochondrial deoxyribonucleic acid; ROS, reactive oxygen species; Sirt6, the name of a nuclear chromatin-bound protein; RAS, renin-angiotensin system; BDNF, brain-derived neurotrophic factor; PKC, the protein kinase C, AGEs, advanced glycation end products; NF-*κ*B, nuclear factor-*κ*B; TNF-*α*, tumor necrosis factor alpha; IL-6, IL-8, interleukins 6 and 8; COX-2, cyclooxygenase 2; ICAM-1, intercellular adhesion molecule 1; MCP-1, monocyte chemoattractant protein 1; VEGF, vascular endothelial growth factor; PHDs, prolyl hydroxylases; HIF-1, hypoxia-inducible factor-1; SCDF-1, stromal cell derived factor-1; RAAS, rennin-angiotensin-aldosterone system; NADPH-oxidase, nicotinamide adenine dinucleotide phosphate-oxidase.

ROS derived from the family of NADPH oxidase (Nox) enzymes may also activate NF-*κ*B and HIF-1 pathways and participate in the development of proliferative diabetic retinopathy. The Nox family, important source of ROS production, consists of seven isoforms named Nox1–5, Duox (dual oxidase) 1, and Duox2 and contributes to vascular injury. Nox1, Nox2, and Nox4 participate in pathological angiogenesis. The RAAS (renin-angiotensin-aldosterone system), and particularly AngII (angiotensin II), is a key stimulator of Nox. RAAS exists in the retina and it is a blockade of AngII and aldosterone attenuates pathological angiogenesis of the retina. However, it is not fully recognized if RAAS has influence on the production of ROS derived from Nox in diabetic retinopathy [170]. The schematic overview of the role of ROS in the development of diabetic retinopathy is presented in Figure 3.

ROS take part in the pathogenesis of cystoid macular edema (CME). CME is caused by inflammatory breakdown of blood-retinal barrier, which results in the accumulation of fluid and protein. Edema and thickening of the macula lead to decrease of vision acuity [174]. In Samanta et al.'s study, both diabetic and normal patients with cystoid macular edema after uncomplicated standardized phacoemulsification surgery exhibited significantly increased activity of ROS determined in the serum samples, in comparison with diabetic and normal patients without CMO after uncomplicated cataract surgery [175].

## 7. The Role of ROS in the Pathomechanism of the Age-Related Macular Degeneration

Age-related macular degeneration (AMD) is the leading cause of permanent, irreversible, central blindness (scotoma in the central visual field makes impossible the following: reading and writing, stereoscopic vision, recognition of colours and details) in patients over the age of 50 in developed countries. It is estimated that approximately 50 million old people suffer from AMD worldwide [176]. The major pathological changes associated with AMD are observed in the functionally and anatomically related tissues including photoreceptors, retinal pigment epithelium (RPE), Bruch's membrane, and choriocapillaris. Soft drusen and/or pigmentary abnormalities are clinically visible symptoms of early AMD. Late (or advanced) AMD occurs in two distinct forms. Visual loss is caused by the “geographic atrophic” death of photoreceptors and retinal pigment epithelium (RPE) cells, so-called “dry-AMD,” GA/AMD, or by formation of the choroidal neovascular membrane (CNV), as a result of pathological angiogenesis, so-called “exudative- or wet-AMD,” CNV/AMD [177, 178].

AMD is a complex chronic neurodegenerative and progressive disease of multifactorial etiology [179]. Advanced age and its related physiological cell apoptosis and tissue involution, together with genetic predisposition and epigenetic modifications, are the strongest risk factors (epigenetics refers to heritable changes in gene expression that do not involve changes to the underlying DNA sequence, a change in phenotype without a change in genotype, and are regular and natural occurrence but can also be influenced by age, the environment/lifestyle, and disease state). However, other factors such as sex and environmental influences such as smoking cigarettes, heart and vascular disorders, hypertension, dyslipidemia/hypercholesterolemia, diabetes, obesity, improper diet, sedentary lifestyle, and phototoxic exposure are also important [179–181].

Excessive ROS production and accumulation together with the oxidative stress seem to play a pivotal role in AMD pathogenesis and RPE cells are critical site of injury in AMD [182]. ROS levels increase in the aging retina, although the retina and RPE cells are rich in both enzymatic and nonenzymic antioxidants. Augmented level of ROS and attenuated antioxidant cell defense systems lead to the oxidative stress and result in damage of photoreceptors, RPE cells, and choriocapillaris in apoptosis process [183, 184].

### 7.1. The Reasons of ROS Accumulation in Outer Part of the Retina in the Course of AMD

The retinal tissue is abundant in ROS, since (i) in the retina is the highest oxygen consumption among all human tissues, (ii) RPE and photoreceptors of the macula are exposed to high-energy light, (iii) the cell membranes of photoreceptors are rich in polyunsaturated fatty acids (PUFA), which are readily oxidized, (iv) there are many photosensitizers in photoreceptors and RPE, and (v) phagocytosis of photoreceptor outer segments (POS) conducted by RPE cells is accompanied by a respiratory burst and rapid eruption of ROS [185].

Photoreceptors are cells of high metabolic activity and high demand for oxygen and nutrients delivered from the blood vessels. Due to the high consumption of oxygen, their supply in the retina is higher than in other tissues [186]. The retina oxygen tension is 70 mmHg [187]. The high partial pressure of oxygen promotes generation of ROS in the retina [185].

Radiation reaching the eye is partly absorbed by the cornea and lens, whereas the rest of it (400–760 nm) penetrates the eye reaching the retina. At the retina level, exposure to visible light simulates RPE cells to phagocytosis (ingestion). The digestion of photoreceptors' outer segments induces formation of superoxide anion in the RPE cells. Epidemiological evidences suggest a direct relationship between phototoxicity (cumulative light exposure) and the development of AMD and susceptibility to the blue light-mediated damage represents one of the aspects of AMD pathogenesis [188]. The blue portion of the visible spectrum of light (441 nm) is dangerous for RPE cells, since it is the most energetic radiation reaching the macula and because it promotes photooxidation of lipofuscin generating the reactive photoproducts including N-retinylidene-N-retinylethanolamine (A2E), DNA oxidation, and cells apoptosis [189, 190]. Blue light leads to disturbances of the outer blood-retinal barrier and damage of POS and alterations in the RPE and choroidal cells are similar to atrophic changes in GA/AMD [191]. Tissues with a high tissue oxygen concentration and a high proportion of membrane lipids are most sensitive to the damage by increased level of ROS and the oxidative stress [192]. Photoreceptors rich in PUFAs are particularly vulnerable to the lipid peroxidation, since the susceptibility of unsaturated fatty acids to oxidation increases with the number of double bonds. The oxidation of PUFAs leads to the development of peroxides and organic radicals [193], as well as other products such as carboxyethylpyrrole (CEP) and 4-hydroxy-2-nonenal (4-HNE), which form adducts with proteins and are accumulated in the outer retina and in drusen [194]. The age-dependent susceptibility of the macula to the lipid peroxidation and its products is connected with the attenuation of antioxidant defense systems with aging. Oxidation of PUFAs lasts many years and leads to the functional and structural impairment of cells membranes and finally to degeneration of photoreceptors [195]. Along with the growth of age, oxidated PUFAs are not efficiently digested in the lysosomes of aged RPE cells and become deposited in the form of lipofuscin. Lipofuscin is a chromophore, serving as the main RPE photosensitizer, which after absorbing a high-energy photon, especially that of blue light, undergoes a variety of photochemical reactions involving ROS formation, which in turn evoke photochemical damage in the retina and RPE cells [196]. A2E is a major hydrophobic fluorophore of RPE lipofuscin, which forms through a multistep biosynthetic pathway, starting with reactions between phosphatidylethanolamine and all-trans-retinal. Upon blue light excitation, A2E acts as a photosensitive generator of singlet oxygen and superoxide, which connect at the carbon-carbon double bonds to form harmful epoxides [197, 198]. A2E-epoxides also accelerate ROS generation and initiate RPE cells damage[199].

The retina is particularly susceptible to aging [1] and vulnerable to the oxidative stress [4], since its two vital components are highly metabolically active and composed of postmitotic cells. Nondividing photoreceptors and RPE cells are particularly prone to the accumulate mtDNA damage due to their inability to reduce defective mitochondria during mitosis. Mitochondria impairment correlates with increased sensitivity of aging RPE cells to the oxidative stress. Changes in mitochondrial number, size, shape, matrix density, cristae architecture, and membrane integrity were more distinct in RPE cells obtained from donors aged 60 and more in comparison with younger individuals. In older donors mitochondria were more elongated, however less numerous [200]. With age, mitochondrial dysfunctions are associated with low ATP level, attenuated mitochondrial membrane potential, reduced cytoplasmic Ca^2+^, and augmented mitochondrial Ca^2+^ sequestration. The decrease level of mitochondrial superoxide dismutase, stimulated long time by the mitochondrial oxidative stress, leads to the increase in superoxide anion, shortening and disorganization of the photoreceptors' outer and inner segments, degeneration of RPE cells, thickening of Bruch's membrane, and finally apoptotic cells death in AMD process [201]. The analysis of the mitochondrial proteomics of RPE cells in advanced stages of AMD showed that the distribution of mitochondrial mutations is qualitatively different in AMD compared to that in normal aging [202, 203].

Chronic low-grade inflammation [204] and hypoxia [205] presented in the aging retina also are the source of ROS production and accumulation.

The products of the oxidative stress trigger chronic low-grade inflammation (pathophysiological parainflammation) process in AMD patients. Pathophysiological parainflammation process mediated by many factors and stimulated by complement system, especially its alternative pathway, and carried out in Bruch's membrane leads to early and advanced CNV/AMD forms. Pathophysiological parainflammation process is connected with the microglial activation carried out in retinal/choroidal interface and leads to the advanced atrophicans AMD form (GA/AMD). Moreover, it is connected with autoantibodies and formation of immune complexes carried out in Bruch's membrane, which leads to the early and advanced AMD, as well as with choroidal macrophages infiltration, which leads to CNV/AMD [204]. ROS impair cells function not only by reacting with nucleic acids, proteins, and lipids but also by inducing production of proinflammatory cytokine [206] and angiogenic signals [207].

In AMD eyes hypoxia is the result of diminished choroidal blood circulation, which confirms measurements of oxygen tension, perfusion pressure, and blood flow rate [208, 209]. The inner part of the retina is better protected from ischemic stress than the outer retina layers, which means that photoreceptors and RPE cells are capable of recovering after an acute hypoxic insult, however not after chronic retinal ischemia and hypoxia, which can lead to cell death and irreversible visual impairment [209, 210]. The retinal blood flow is disturbed in both dry and CNV/AMD type [208]; the reduction in choroidal perfusion has been positively correlated with the disease progression [205]. During inflammation, hypoxia in the retinal cells may result from increased consumption of oxygen due to the increased metabolic activity of the inflamed retina [205].

Superoxide anions are involved in the regulation of cells adaptation to hypoxia via HIF-1*α* factor [211] and are involved in the regulation of mitochondrial autophagy process [212, 213].

Chronic elevated ROS levels and the oxidative stress, pathophysiological parainflammation, and long stay hypoxia decrease the ability of RPE cells to remove damaged or nonfunctional proteins via the lysosomal clearance system, including macroautophagy [214]. In aged RPE cells the substrate for autophagy is degraded by lysosomal acid hydrolases, including cathepsins D, B, and L, after autophagolysosome, and Rab7, LAMP-2A, and SNAREs proteins are critical for the fusion of lysosome and autophagosome. Ubiquitin (Ub), LC3II, and p62 complexed to the substrate connect autophagy with the proteasomal clearance system [214].

A marked reduction of macroautophagic activity with aging has been associated with an increase in chaperone-mediated autophagy [215]. Experimental studies confirm that ROS also take part in microautophagy and disturb endothelial reticulum (ER) in AMD process. Human RPE cells exhibited in vitro ROS accumulation and subsequent elevation of GRP78 and CHOP expression (indicators of ER stress) after A2E and blue light-induced damage. Moreover, N-acetylcysteine (NAC), ROS scavenger, diminished expression protein of ER stress [216]. In another study, t-butylhydroperoxide induced the oxidative stress which led also to the accumulation of ROS in the internal space (lumen) of the endoplasmic reticulum and disturbed ER homeostasis in RPE cells [217].

**Figure 4:**
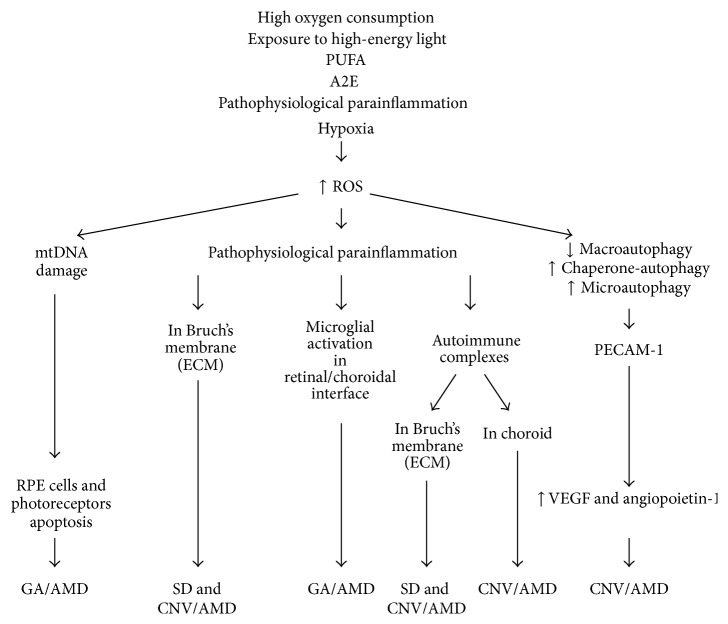
Schematic overview of the ROS influence on the development of early and advanced forms of age-related macular degeneration. PUFA, polyunsaturated fatty acid; A2E, a component of retinal pigmented epithelial cell (RPE) lipofuscin; ROS, reactive oxygen species; mtDNA, mitochondrial deoxyribonucleic acid; ECM, extracellular matrix; PECAM-1, platelet endothelial cell adhesion molecule; VEGF, vascular endothelial growth factor; SD, soft drusen; GA/AMD, geographic atrophy/age-related macular degeneration; CNV/AMD, choroidal neovascularization/age-related macular degeneration.

According to Blasiak et al., triplet consists of the oxidative stress, hypoxia, and autophagy which play an important role in CNV/AMD pathogenesis [214]. Pathological angiogenesis in AMD is connected with the activity of VEGF and angiopoietin-1 (and its receptor) [218]. Two proteins, that is, platelet endothelial cell adhesion molecule (PECAM-1) and thrombospondin-1 (TSP-1), act as linkage molecules, which have a reciprocal relationship with autophagy and angiogenesis mediated by angiopoietin 1 [214]. TSP-1, a common denominator between autophagy, angiogenesis, and AMD, acts as antiangiogenic molecule (and a target for antineovascular therapy). Impaired expression of thrombospondin-1 in Bruch's membrane and choroidal vessels was shown in the rodent eyes with age-related macular degeneration [219]. The schematic overview of the role of ROS in the development of early and advanced AMD is presented in Figure 4.

## 8. Conclusions

Excessive production of the reactive oxygen species and the oxidative stress play important role in the pathogenesis of many age-related ocular diseases and other pathologies of the anterior and posterior eye segment in adults.

ROS stimulate cells' death via apoptosis process, participate in the activation of proinflammatory and proangiogenic pathways, and are associated with the autophagy process.
